# Three-Dimensional Printing Strategies for Irregularly Shaped Cartilage Tissue Engineering: Current State and Challenges

**DOI:** 10.3389/fbioe.2021.777039

**Published:** 2022-01-05

**Authors:** Hui Wang, Zhonghan Wang, He Liu, Jiaqi Liu, Ronghang Li, Xiujie Zhu, Ming Ren, Mingli Wang, Yuzhe Liu, Youbin Li, Yuxi Jia, Chenyu Wang, Jincheng Wang

**Affiliations:** ^1^ Orthopaedic Medical Center, The Second Hospital of Jilin University, Changchun, China; ^2^ Department of Plastic and Reconstructive Surgery, The First Hospital of Jilin University, Changchun, China

**Keywords:** 3D printing, cartilage tissue engineering, irregularly shaped cartilage, shape imitation, bionic performance

## Abstract

Although there have been remarkable advances in cartilage tissue engineering, construction of irregularly shaped cartilage, including auricular, nasal, tracheal, and meniscus cartilages, remains challenging because of the difficulty in reproducing its precise structure and specific function. Among the advanced fabrication methods, three-dimensional (3D) printing technology offers great potential for achieving shape imitation and bionic performance in cartilage tissue engineering. This review discusses requirements for 3D printing of various irregularly shaped cartilage tissues, as well as selection of appropriate printing materials and seed cells. Current advances in 3D printing of irregularly shaped cartilage are also highlighted. Finally, developments in various types of cartilage tissue are described. This review is intended to provide guidance for future research in tissue engineering of irregularly shaped cartilage.

## 1 Introduction

Cartilage is widely distributed throughout the human body, and is mainly composed of extracellular matrix (ECM) with embedded chondrocytes (Anderson, 1962). The morphologies of the auricle, nose, trachea, and meniscus are irregular. The shape of cartilage varies, often forming irregular arcs or circular patterns depending on its function. These functions include maintaining the specific shape of a tissue or buffering mechanical forces that deform tissues during movement (Honkanen et al., 2016; Zhou et al., 2018). Due to a lack of blood perfusion, cartilage cannot be repaired as easily as other injured structures such as skin and bone that contain blood vessels. Current strategies for restoring damaged cartilage that is irregularly shaped have not met the initially high expectations (Le et al., 2020). For 3D printing of irregularly shaped cartilage, the challenges are specific to each structure.

For repair of auricular and nasal cartilage where aesthetic appearance is the primary consideration, modified autologous costal cartilage is commonly used for transplantation (Foerster, 1966a; Vuyk and Adamson, 1998). However, autologous transplantation may cause damage to the donor site, and patients are often unsatisfied with the appearance of the reconstructed area. In addition, there is a risk of postoperative complications such as infection (Thorne et al., 2001; Yamada, 2018). As an alternative to autologous implantation, commercial auricular prostheses are also a rational choice as they involve pre-assembled scaffolds with C- and Y-shaped frames made of composite porous polyethylene (Medpor) material (Cenzi et al., 2005). However, surgical implantation is often accompanied by complications like erosion, infection, absorption collapse, inflammation, and displacement (Younis et al., 2010). Furthermore, this treatment is only applicable to total replacement of the auricular scaffold, which is not appropriate for repair of local auricle defects (Yamada, 2018).

Nasal prostheses are not only used in repair of nasal cartilage defects caused by congenital diseases and trauma but also in rhinoplasty. Nasal septal cartilage, auricular cartilage, and alar cartilage are often used as grafts during autogenous cartilage transplantation. Bone grafting can also be used in rhinoplasty, but it is important not to ignore the side effects of rhinoplasty or complications such as open roof, stair-step, and rocker deformities, bony pyramid and nostril asymmetries, and limited donor sites (Vuyk and Adamson, 1998). The trachea and meniscus function in load bearing and supporting, which is difficult to restore following injury due to a lack of blood vessels (Fabre et al., 2013). In this case, the size and shape of the implanted scaffold can be customized with the aid of software according to the patient’s needs. In addition, incorporation of acellularized hydrogels into a scaffold can promote biocompatibility after implantation (Mouser et al., 2020).

**SCHEME 1:**
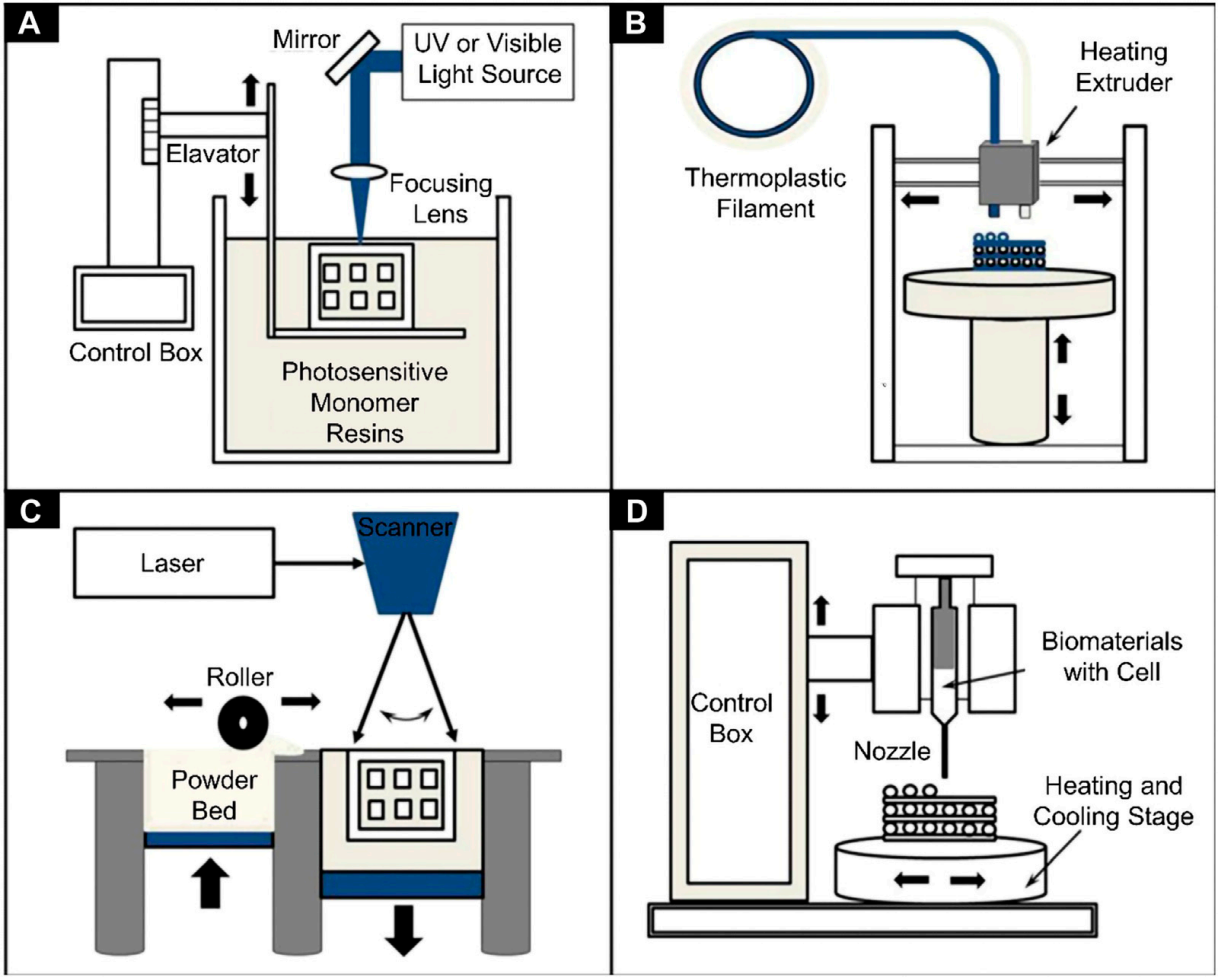
Schematics of different 3D bioprinters used in tissue engineering. **(A)** Vat photopolymerization, **(B)** Fused filament fabrication, **(C)** Selective laser sintering, and **(D)** Inkjet 3D printing (Gu et al., 2016).

Taken together, the lack of suitable repair materials of proper morphology, hardness, and biocompatibility is a major problem in cartilage defect repair, and 3D printing has come to the forefront of cartilage tissue engineering to address these problems. In 1986, 3D printing technology was introduced to the public (Hull, 1996). Among 3D printing technologies, fused deposition modeling (FDM) involves melting a polymer so that it flows through a nozzle, allowing the scaffold to be suitably shaped as it is printed layer by layer (Penumakala et al., 2020). In order to construct 3D tissues, 3D bioprinting was developed to build complex structures by incorporating cells and growth factors into a hydrogel and extruding the composite material layer by layer according to a pre-designed 3D model (Gu et al., 2016; Fenton et al., 2019; Mouser et al., 2020) (Scheme 1). In addition to precise control of scaffold shape, spatial resolution and mechanical properties can be controlled during 3D printing (Mouser et al., 2017).

**SCHEME 2:**
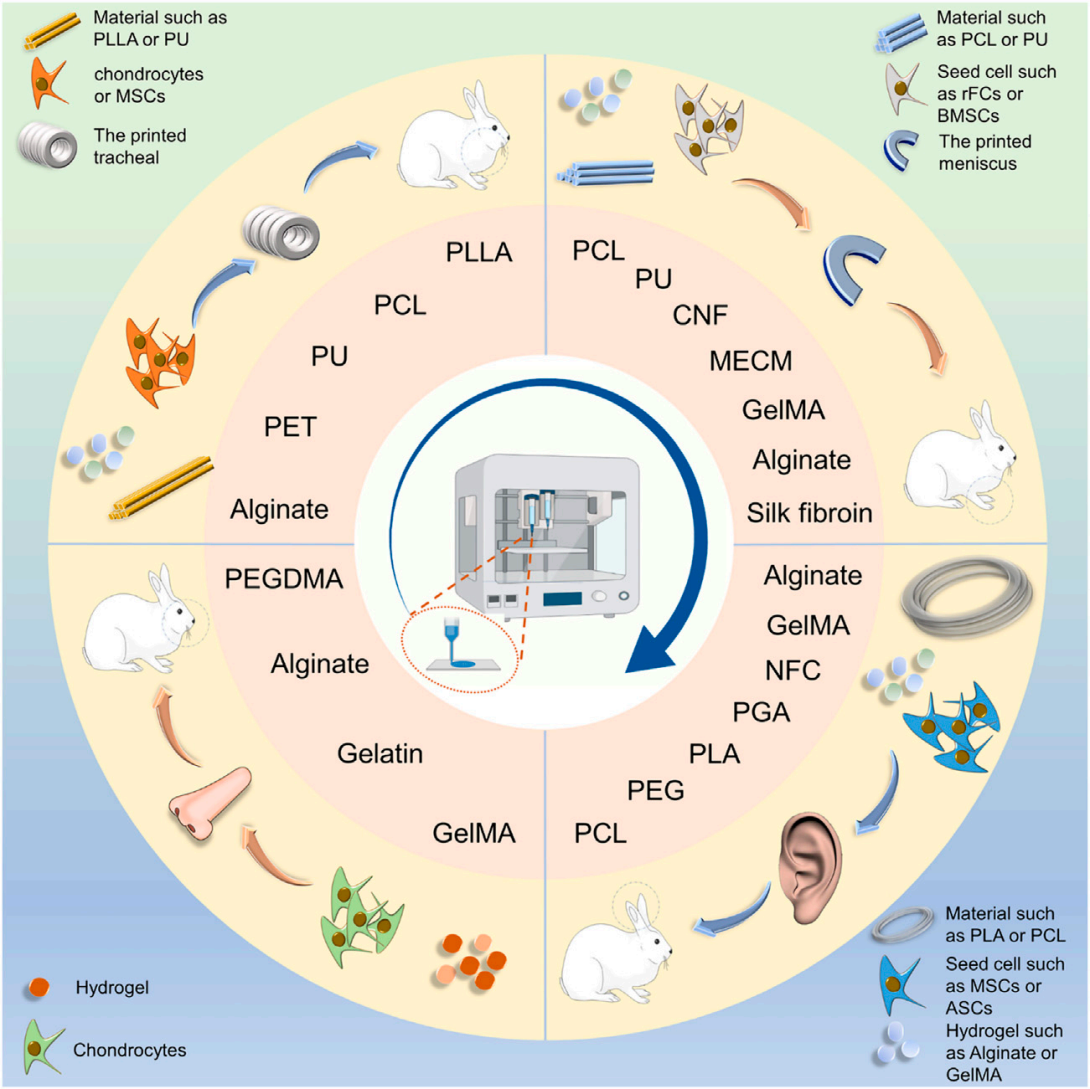
Schematic illustration of scaffold fabrication for cartilage engineering of the auricle, nasal cartilage, trachea, and meniscus. Polymers and hydrogel were made into irregularly shaped cartilage scaffolds using 3D printing technology. PLLA, poly(L-lactic acid); PCL, Poly(ε-caprolactone); PU: Polyurethane; PEG, polyethylene glycol; PEGDMA, polyethylene glycol dimeth-acrylate; PGA, polyglycolic acid; NFC, nanofiber cellulose; MECM, meniscus extracellular matrix.

This review summarizes studies of 3D printing of irregularly shaped cartilage scaffolds and discusses the current status of that research, including the use of common materials, cells, and related 3D printing technologies (Scheme 2). The intend of this review is to provide guidance for future research on irregularly shaped cartilage in tissue engineering.

## 2 Materials Used for 3D Bio-Printed Irregularly Shaped Cartilage

3D printing technology has the potential to fundamentally enhance regenerative medicine (Rajabi et al., 2021). Several studies of 3D printing of irregularly shaped cartilage have reported the use of high molecular weight polymers, including poly (lactic-co-glycolic acid) (PLGA) (Wei P. et al., 2020), poly (lactic acid) (PLA) (Rosenzweig et al., 2015), poly (ε-caprolactone) (PCL) (Xu et al., 2019; Li et al., 2021), and polyurethane (PU) (Kim H. Y. et al., 2019), to print cartilage scaffolds that are stable due to their optimal mechanical properties. In addition, depending on the experimental requirements, hydrogels with good biocompatibility such as silk fibroin (SF) (Rosadi et al., 2019), alginate, gelatin (Yang et al., 2019), and chitosan (CS) are often chosen as printing materials or used as part of a cartilage scaffold. Such hydrogels can act as a cell matrix to support cell growth (Unagolla and Jayasuriya, 2020). The following sections describe each material in detail.

### 2.1 Synthetic Macromolecular Polymer Materials

#### 2.1.1 Poly(Lactic-co-Glycolic Acid)

PLGA possesses ideal bio-compatibility and bio-degradation properties. PLGA is approved by the U.S. Food and Drug Administration for clinical applications, including drug synthesis and prosthesis fabrication (Gentile et al., 2014). PLGA is a type of thermoplastic, and its melting temperature is 190°C, which eliminates the possibility of printing PLGA with living cells (Kim et al., 2016). The characteristics of PLGA are determined by its molecular weight, the type of end caps, and the lactic acid (LA)/glycolic acid (GA) ratio. PLGA with an ester end cap is more stable during printing and degrades more slowly. In contrast, PLGA degradation is accelerated by an acid end cap. PLGA viscosity increases with increasing LA/GA ratio. Hence a PLGA scaffold with a higher LA/GA ratio and ester end cap is more suitable for preparation of cartilage scaffolds (Guo et al., 2017; Gradwohl et al., 2021). In addition, PLGA is also used to make electrospun scaffolds. Due to the bridging and pull-out properties of PLGA, materials mixed with PLGA may exhibit ductile fracture during bioprinting (Zhao G. et al., 2020).

#### 2.1.2 Poly(ε-caprolactone)

PCL is a polymer obtained by ring-opening polymerization of ε-caprolactone monomers (Bhattacharjee et al., 2021). The compressive modulus of pure PCL is approximately 4.5 MPa, which is significantly lower than that of PLA and other materials (Popescu et al., 2018). Nevertheless, the elastic modulus of the PCL is about 30 MPa, which is closer to that of native cartilage and makes it more suitable for 3D printing of cartilage (Zhang et al., 2017). The low melting point of PCL (55°C) renders it easier to extrude in 3D printing (Dong et al., 2017). Owing to a low glass transition temperature and high thermal stability, PCL has great potential for tissue engineering (Chia and Wu., 2015). PCL can be fabricated into a scaffold by selective laser sintering (SLS) (Patel et al., 2015) and direct ink writing (Zhang et al., 2020). Because high temperature can damage cells, PCL with a lower melting point is more appropriate for acting as a protective layer on bioactive scaffolds (Kim et al., 2016).

#### 2.1.3 Poly(Lactic Acid)

PLA is a type of thermoplastic polymer made from natural renewable resources, and is biodegradable (Wang et al., 2015). PLA can be processed into orthopedic implant materials by FDM, SLS stereolithography (SLA), and other 3D printing technologies (Van den Eynde and Van Puyvelde, 2018; Chen X. et al., 2019). When the temperature rises to 41.5°C, the DNA of cells will be damaged and the cells will die (Akihisa et al., 2004). The high temperature required for printing PLA harms cells seeded onto the surface of the scaffold, but printing PCL between two layers of PLA will make the cell mortality reduce since the melting point of PCL is 60°C. Heated PLA is continuously extruded into three layers by a nozzle, and layers of PCL are extruded between them. Cells encapsulated in sodium alginate are then deposited in the resulting space (Kim et al., 2016). Moreover, printing of scaffolds using layer-by-layer printing technology can improve survival of cells in hydrogel scaffolds (Baena et al., 2019).

When PLA and PCL are used together to prepare 3D-printed scaffolds, not only is cell damage reduced but scaffold mechanical properties are improved. When a PLA mesh covers the surface of a PCL scaffold, its strength significantly increases (Pensa et al., 2019). PLA can be added to a variety of polymers used in 3D-printing. It can also be modified to form poly (L-lactic acid) (PLLA) and poly (D-lactic acid) (PDLA) polymers by incorporating enantiomeric L- and D-lactic acid units, respectively (Masutani and Kimura, 2017). In addition, the toughness of PLLA can be increased by adding PCL to form poly (l-lactide-co-e-caprolactone) (PLCL) (Liu W. et al., 2020).

#### 2.1.4 Polyurethane

PU is usually made from a combination of diisocyanate and polyol (Wang P. et al., 2019), and can be divided into two forms: thermosetting and thermoplastic (Griffin et al., 2020), both of which have good biocompatibility. Compared with other materials, PU has higher elasticity and tensile strength (Griffin et al., 2020). In addition, PU is thermally sensitive and exhibits a sol-gel transition at 37°C (Hsieh and Hsu., 2015). As a result, it has been used to fabricate scaffolds in some soft tissue engineering experiments (Luo K. et al., 2020; Farzan et al., 2020). PU is widely used in various fields of 3D printing by way of fused fiber fabrication (FFF), bio-plotting, SLA (Griffin et al., 2020), and FDM (Xiao and Gao., 2017). The tensile strength and break elongation of thermoplastic polyurethane (TPU) scaffolds printed by FDM can reach 46.7 MPa and 702%, respectively, by arranging the fiber at a 45° angle and forming the scaffold at 215°C (Xiao and Gao., 2017). 3D-printed scaffolds made of PU have excellent shape memory. In addition, they are biodegradable and osteogenic (Wang et al., 2018). Moreover, PU has been used in research on 3D printing of tendons. Scaffolds have been successfully printed by combining the advantages of PU and PCL (Merceron et al., 2015).

### 2.2 Natural Materials

#### 2.2.1 Silk Fibroin

SF is a natural material extracted from silkworm cocoons. It is biodegradable and is widely used in medical fields. To date, SF has been used with 3D printing technology to repair defects in skin, bone, cartilage, and vascular tissues (Wang Q. et al., 2019). Pure SF has relatively poor mechanical strength, so it is often mixed with other materials such as hydroxypropyl methylcellulose, gelatin, PEG, and glycerol to improve its properties (Mu et al., 2020). The mixture of SF and hydroxy propyl methyl cellulose of methacrylate (HPMC-MA) has excellent mechanical properties that depend on methacrylate content. The compressive stress of the mixture is 25 KPa (Ni et al., 2020). In addition, the mechanical properties of silk fibroin scaffolds were improved by the technology of biomineralization and pre-mineralization (Neubauer et al., 2021). Biomineralization is the process of metabolizing cells to form minerals (Neubauer and Scheibel, 2020). Graphene oxide, β- Tricalcium phosphate, and nano hydroxyapatite can be added to silk fibroin fibers to pre-mineralize silk fibroin (Liu F. et al., 2020; Wang L. et al., 2020; Zafar et al., 2020). Except for the improvement of mechanical properties, the viscosity of the mixture made of silk fibroin and gelatin will be improved after tyrosinase induced crosslinking (Chameettachal et al., 2016).

In addition to common chemical cross-linking methods, SF can also be cross-linked by physical methods to improve its mechanical properties (Chi et al., 2018). Cartilage acellular matrix (CAM) has properties suitable for cell growth, but its extrusion properties are unsuitable for printing. The addition of SF endows CAM with a fluidity that makes it suitable as a bio-ink for 3D printing (Chi et al., 2018). A solution with useful nanostructural and mechanical properties can be prepared by dissolving SF in a CaCl_2_ formic acid solution. The maximum elastic modulus and final tensile strength of the stretched SF film in the dry state are 4 GPa and 106 MPa, respectively (Zhang et al., 2015).

#### 2.2.2 Alginate

Alginate can be used in various fields, including wound healing, drug delivery, and tissue engineering (Liu et al., 2019; Wei X. et al., 2020; Oveissi et al., 2020; Khoshnood et al., 2021). Alginate is usually extracted from brown algae and possesses excellent biocompatibility. Ba^2+^ or Ca^2+^ salts can rapidly cross-link sodium alginate sol into a gel state (Zhang et al., 2019). It has been shown that cell activity in a ring scaffold cross-linked by BaCl_2_ is stronger than when cross-linked by CaCl_2_ (Dranseikiene et al., 2020). Cross-linking alginate can also increase its mechanical strength, which provides it with many applications in tissue engineering. Alginate gel is commonly used in 3D printing as it has favorable rheological properties (Kim M. H. et al., 2019). Sodium alginate gels mixed with gelatin to form a hybrid bio-ink possess improved viscosity and elasticity (Cheng et al., 2019; Liu et al., 2019; Soltan et al., 2019). The low degradation rate of alginate gel scaffolds make them poor implants for tissue regeneration. However, using gamma rays to change the molecular weight distribution can accelerate degradation of sodium alginate gels (Kong et al., 2004).

The mechanical strength of an alginate gel is quite low. Mixing PEG and sodium alginate produces a gel with high mechanical strength that is suitable for growth of human bone marrow mesenchymal stem cells (Melo et al., 2019). The rheological properties of alginate gel-based inks influence the shape fidelity and resolution of the printed structure, and they can be improved by mixing alginate gel with carrageenan hydrogels (Kim M. H. et al., 2019). A set of experimental studies has shown that the rheology (viscosity) of the bioprinting ink formed by mixing alginate gel with gelatin is dependent on the printing temperature (Liu et al., 2019). For cells mixed into the hydrogel, adhesion to the gel polymer is essential, and alginate gel modified by dopamine can significantly strengthen cell adhesion (Luo et al., 2019).

#### 2.2.3 Gelatin

Gelatin is a water-soluble and biodegradable polypeptide produced by hydrolysis of collagen. Its enzyme degradation rate is unsatisfactory and it has poor mechanical stability, which limits its applications in biological tissue engineering (Raucci et al., 2019). However, combining gelatin with other materials can improve its viscosity and make it more suitable for 3D printing (Duan et al., 2013; Unagolla and Jayasuriya, 2020). Bio-inks containing gelatin exhibit good fluidity at 35°C, which is conducive to extrusion during 3D printing. After cooling down on the casting platform at 4°C, the scaffold solidifies rapidly (Dutta et al., 2021). In cartilage tissue engineering, MMP2 activity can be induced by gelatin to degrade the synthetic matrix. Then a pericellular zone is formed to accumulate extracellular matrix growth factors and the newly synthesized matrix, so as to the differentiation of chondrocyte is promoted (Chameettachal et al., 2016).

#### 2.2.4 Chitosan

CS is prepared by alkaline deacetylation of chitin. CS has many desirable properties such as natural non-toxicity, histocompatibility, biodegradability, and antibiosis (Long et al., 2019). A fluid suitable for extrusion printers can be prepared by dissolving CS in acid solutions such as GA and LA. A solution made by dissolving CS in 30 wt% GA is suitable for preparation of CS catheters and 3D printing as its tensile strength and Young’s modulus are 10.98 ± 0.61 MPa and 12.38 ± 1.19 MPa, respectively (Zhao C. Q. et al., 2020). CS can also be used to improve the performance of other scaffolds. Adhesion of calcium phosphate to PLA can be increased by adding a layer of CS gel to a PLA scaffold, which also improves the mechanical properties of the scaffold (Schneider et al., 2018). Combining CS with mixtures that contain PLA can make the material more hydrophilic (Cheng and Chen, 2017). CS mixed with saline alginate gel can increase its viscosity, making it more suitable for 3D printing (Liu et al., 2018).

## 3 Seed Cells Used for 3D Bio-Printed Irregularly Shaped Cartilage

Depending on the specific requirements, the best biomimetic effect can be achieved by including materials such as seed cells (Kačarević et al., 2018; Zhang and Song, 2018), and selection of an optimal seed cell is of vital importance to achieve cartilage regeneration. Chondrocytes and mesenchymal stem cells (MSCs) are commonly used in cartilage tissue engineering research.

Chondrocytes are often chosen as seed cells, but the tissue sites for obtaining them are limited and vulnerable to injury. Chondrocytes used in cartilage tissue engineering have low immunogenicity, but isolation methods are complex and less well developed than those for MSCs (Francis et al., 2018a)—and considering their multi-directional differentiation potential, MSCs are often preferred (Aggarwal and Pittenger., 2005). In addition, MSCs can inhibit inflammation in scaffolds implanted *in vivo*, and reduce damage resulting from foreign body reactions (Ding et al., 2016).

Some types of MSCs can differentiate toward cartilage cells. Among types of MSCs, bone marrow stem cells (BMSCs) are commonly used in cartilage tissue engineering. As the first identified mesenchymal stem cells, there are many studies on BMSCs (Strioga et al., 2012). BMSCs have the advantage of being plentiful and easy to obtain (Wang Y.-H. et al., 2020), and they have the ability to differentiate toward ecto-, meso-, and endodermal cell lineages, including adipocytes, germ cells, chondrocytes, osteoblasts, pancreatic islet-like cells, hepatocytes, myocytes, annulus fibrosus-like cells, and neuron-like cells (Li et al., 2018). However, BMSCs collected from the elderly are not entirely suitable owing to their limited ability to differentiate and low rate of proliferation (Pittenger et al., 1999; Zaim et al., 2012). There is still a great deal of controversy concerning selection of stem cells. Compared with BMSCs, adipose-derived mesenchymal stem cells (ADSCs) are more plentiful and easier to obtain (Ra et al., 2011; Jo et al., 2014). ADSCs have the potential to differentiate into mesoderm tissue lineages, including bone, cartilage, fat, and muscle (Francis et al., 2018b). Studies have shown that ADSCs are more likely to differentiate into cartilage than are BMSCs (Jang et al., 2015). Umbilical cord blood mesenchymal stem cells (UCB-MSCs) have a greater ability to differentiate into cartilage and express proteins and cytokines than do BMSCs (Lo et al., 2013). However, using BMSCs as seed cells is more conducive to collagen formation and cartilage repair (Contentin et al., 2019). Nevertheless, these conclusions are influenced by the different evaluation criteria used, and therefore the choice of MSC depends on the purpose of the experiment. It has been shown that a mixture of ADSCs and chondrocytes can be used for seeding cells onto a biodegradable scaffold (Morrison et al., 2018).

## 4 3D-Printed Irregularly Shaped Cartilage Scaffolds

The structural complexity of irregularly shaped cartilage increases the difficulty of manufacturing scaffolds. However, the emergence of 3D printing technology provides a new solution for repair of irregularly shaped cartilage. On this basis, and considering the biocompatibility of materials and selection of seed cells, useful improvements have been made that are adapted to the specific requirements of different structures to facilitate study of 3D printing of irregularly shaped cartilage. This section summarizes progress in 3D printing technology used for reconstruction of four types of irregularly shaped cartilage: auricular, nasal, tracheal and meniscus.

### 4.1 3D-Printed Auricular Cartilage Tissue

Developments in medical procedures and knowledge have facilitated surgical repair or improvements of features that affect facial beauty. There is a long history of research on auricular repair, including treatment of mild injuries such as earlobe and earring injury, and severe injuries such as when the entire auricle is bitten or cut (Song et al., 2020). Because the auricle is an important facial feature, congenital auricle deformity or injury can damage mental health. 3D-printed auricular cartilage can be used for auricle shape reconstruction. The first step in 3D printing of an auricle is to design its shape, and one of two methods involving CAD software is typically used: either import DICOM files to synthesize auricle shape, or design the required graphics directly (Zhou et al., 2018). The resulting auricle model then needs to be converted into STL (structured template language) form. An iTOP (integrated tissue and organ printer) can also be used to print the ideal ear shape (Kang et al., 2016). Currently there are two main types of 3D-printed auricle scaffold. In the first, the auricle shape is printed directly using hydrogel, and in the second, the support structure of the auricular scaffold is first printed using biomaterials, and then the surface of the scaffold is covered by a cell-containing hydrogel using 3D printing or immersing the scaffold into the hydrogel (Jang et al., 2020).

#### 4.1.1 Preparation of Auricular Scaffolds Using Hydrogel

Because of the pretty biocompatibility of hydrogels, some studies have been devoted to research on 3D printing of auricular scaffolds using hybrid hydrogels. Bio-ink produced by mixing gelatin with SF from two sources (*Philosamia ricini* and *Bombyx mori*) can be used to print auricular cartilage as it gels without cross-linking (Singh et al., 2019), which eliminates potential side effects of the cross-linker. This material shows excellent fidelity, stability, swelling properties, biodegradability, and promotes cell viability (Singh et al., 2019). In addition, a hybrid bio-ink can be photocured during 3D printing by adding methacrylic anhydride and a photoinitiator in a mixture of gelatin and hyaluronic acid. Scaffold degradation becomes prolonged after being freeze-dried, and the compression strength of lyophilized scaffolds is significantly greater than for other scaffolds. This cross-linking method allows sufficient time for preparation of composite materials and development of chondrocytes (Xia et al., 2018). Although freeze-drying results in some improvement in the mechanical properties of hydrogel scaffolds, it remains to be seen whether they remain stable as the gel degrades.

#### 4.1.2 Preparation of Auricular Scaffolds With Polymer Materials and Hydrogel

The other kind of 3D-printed auricular cartilage is that made of composite polymer materials and hydrogel. Polymers such as PLA, PCL, and hydrogels are often combined to fabricate auricular scaffolds (Table 1). PCL material is often preferred for auricular cartilage scaffold printing because of its high compression modulus (Olubamiji et al., 2016). The compression modulus of the scaffold is affected by the properties of the material and the diameter of the scaffold, which are related to printing speed. For a given flow rate through the printer nozzle, the higher the printing speed, the smaller the diameter of the extruded stream, which alters the mechanical properties of the printed material. The compression modulus of the PCL scaffold decreases with increasing nozzle diameter, and its flexibility is affected by line spacing and angle. Running the 3D printer nozzle along the 0°/45° direction to print the scaffold will make the scaffold more flexible (Chung et al., 2020). Experimental studies have shown that scaffolds printed with PCL and hydrogel mixed with cells are more conducive to cartilage formation than if the cells are placed on the surface of the composite scaffold (Park et al., 2017).

**TABLE 1:** Summary of 3D-printed auricular cartilage.

Material	Seed cells	Bioprinting Technology	Key point	References
Nanofibrillated cellulose (NFC)/Alginate hydrogels	Human nasoseptal chondro-cytes (hNC)	Extrusion printing	The hybrid bio-ink, mixed at an 80:20 ratio of NFC to alginate, is printed by an extrusion printer	Markstedt et al. (2015)
PCL/PEG/Alginate hydrogels	Human adipose derived stem cells (ASCs)	Extrusion printing	PEG and PCL were used as the sacrificial and main materials of the framework	Lee et al. (2014)
PCL/methacrylate Gelatin (GelMA)/Hyaluronic acid (HAMA)/Pluronic F-127 hydrogel	Bone marrow-derived human (BMSCs) Mesenchymal stem cells (MSCs)	Extrusion printing	PCL and GelMA-HAMA were used for hybrid printing, Pluronic F-127 was selected as a sacrificial material	Chung et al. (2020)
Type I collagen gel/PCL	Porcine auricular cartilage	EOS P100 laser sintering system	The cells were embedded in collagen I gel solution and the cell suspension was then pipetted into the PCL scaffolds	Zopf et al. (2015)
PU	Tonsil-derived mesenchymal stem cells	—	3D printing of auricular cartilage scaffold was performed with PU material	Kim et al. (2019a)
PCL	—	Fused deposition system	Creating auricular model and 3D printing with PCL material	Jung et al. (2019)
PCL/PGA/PLA	Picrotia chondrocytes (MCs)	Fused deposition system	The auricular scaffold used PCL as an inner core, which was wrapped with PGA unwoven fibers and coated with PLA.	Zhou et al. (2018)
Gelatin/fibrinogen/hyaluronic acid (HA)/glycerol/PCL/Pluronic F-127 hydrogel	3T3 fibroblasts	An integrated tissue–organ printer (ITOP)	3T3 cells were mixed in the prepared hydrogel, the auricular scaffold structure was printed simultaneously with PCL, and Pluronic F-127 hydrogel was used to print the sacrificial layer to maintain scaffold structure	Kang et al. (2016)

**FIGURE 1:**
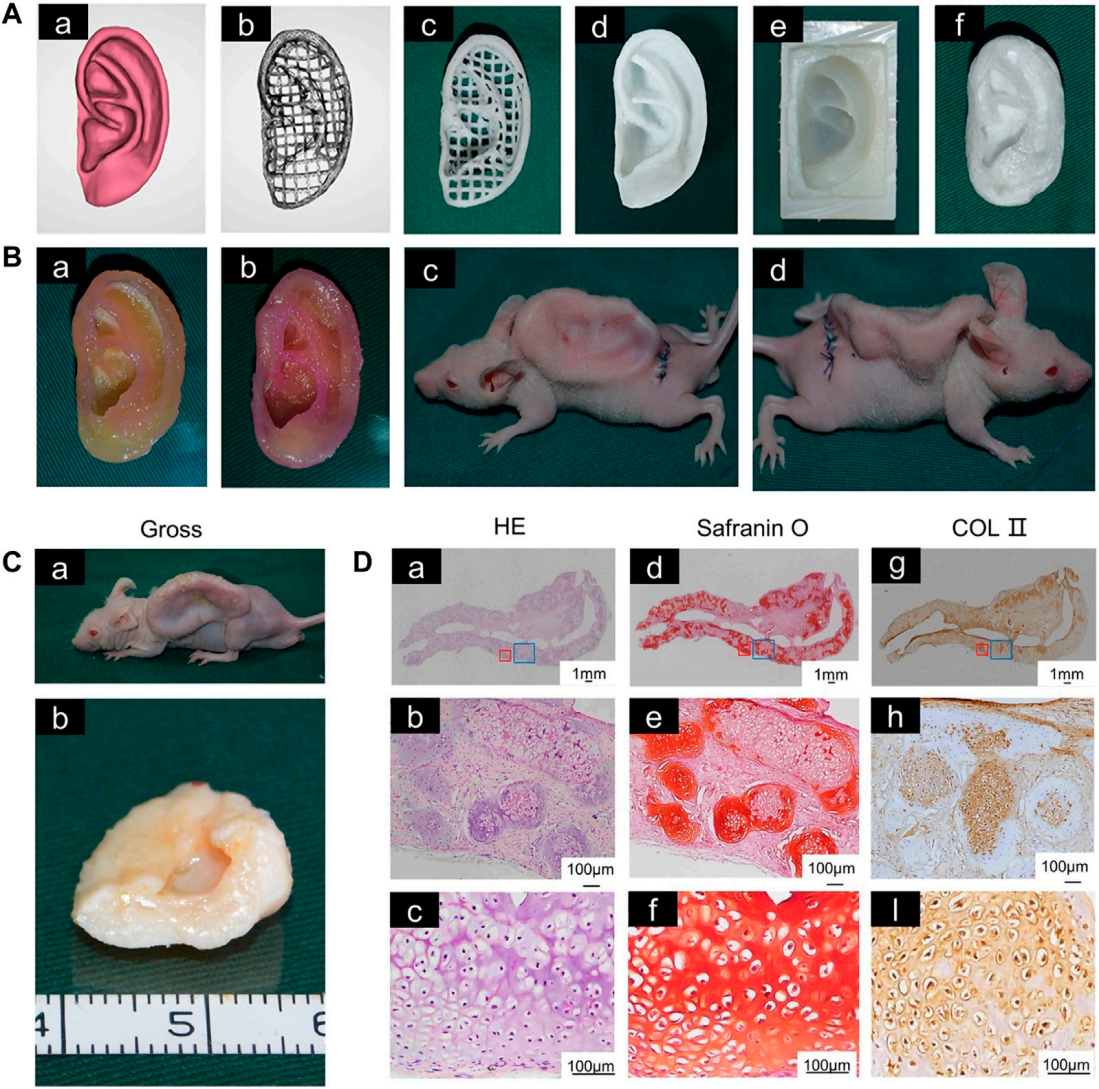
3D-printed auricular scaffold and *in vivo* experiments. **(A)** Design of a 3D-printed auricle model and the resulting printed scaffold; **(a)** 3D digital model of the human auricle, **(b)** 3D mesh digital model, **(c)** PCL inner core, **(d)** human auricle-shaped PCL positive mold, **(e)** silicone casting mold, and **(f)** ACM/Gelatin-PCL scaffold. **(B)** Auricular scaffold before implantation and after implanting it into a nude mouse for 2 weeks. **(C)** Images of scaffolds cultured *in vivo* for 12 weeks. **(D)** H&E and safranin-O staining showed lacunar structure and cartilage-specific ECM deposition (Jia et al., 2020).

For the auricular scaffolds printed with PCL, the shape of the pores in the scaffold can affect the cells seeded into it. Spherical pore design not only facilitates chondrocytes adopting a shape characteristic of natural cartilage *in vivo* but also accelerates matrix deposition (Zopf et al., 2018). However, incorporation of hydrogel into scaffold pores cannot guarantee the desired cell content. Scaffolds with a sandwich structure can to some extent reduce the loss of hydrogel and cells. Cell survival has been promising in alginate hydrogels placed between two printed PCL scaffolds to form a sandwich structure for auricle implantation. This new method of constructing auricular cartilage considers both mechanical properties and cell viability (Visscher et al., 2019). When selecting a hydrogel loaded with cells, adding an acellular cartilage matrix (ACM) to the gelatin can produce a hydrogel more similar to that *in vivo*, which is conducive to cell growth (Jia et al., 2020) (Figure 1).

Covering a PCL scaffold with PGA has been shown to be beneficial to auricular chondrocytes. The scaffold remained intact and chondrocytes differentiated into cartilage 2.5 years after implantation. This study supported the feasibility of 3D-printed cartilage tissue for clinical applications (Zhou et al., 2018). For 3D printing, in addition to scaffold stability and cell survival in hydrogels, it is also necessary to consider the rheological and cross-linking properties of hydrogels. Bio-ink prepared by mixing nanofiber cellulose (NFC) with sodium alginate is promising for bio-printing because of the shear-thinning effect. Moreover, the hybrid material simultaneously offers a dual-cross-linking pattern and greater storage modulus (Markstedt et al., 2015). However, further *in vivo* and clinical trials of hybrid materials are still required to verify their efficacy. On this basis, bacterial nano-cellulose (BNC) was modified by a water-based anti-collision agent, and the Iβ phase of BNC was replaced by the Iα phase, which was more thermodynamically stable. Higher water retention and stronger mechanical properties were obtained in this way (Apelgren et al., 2019). Studies of the feasibility of tissue engineered cartilage have been carried out in animals. As early as 1997, a group of researchers implanted PLGA-PLA structure into mice lacking a thymus, which confirmed the feasibility of auricle construction using chondrocytes and biodegradable polymers (Cao et al., 1997).

#### 4.1.3 3D-Printed Auricular Cartilage With a Sacrificial Layer

**FIGURE 2:**
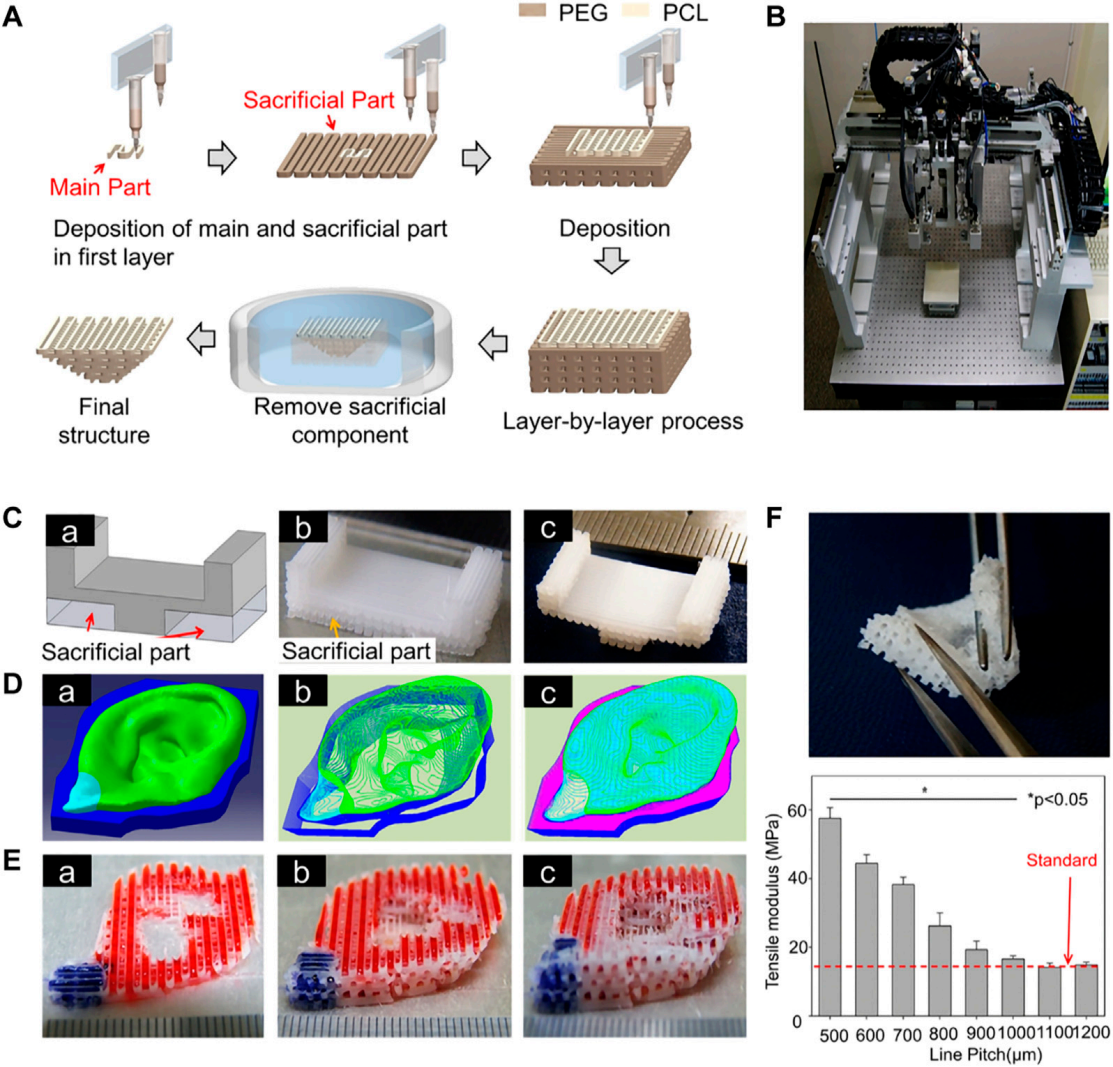
3D-printed auricular scaffold using sacrificial layer technology. **(A)** Fabrication of inverse porous pyramid structure employing sacrificial layer technology. **(B)** Schematic diagram of the multi-head tissue/organ building system (MtoBS). **(C)** Overhanging structure (20 mm × 10 mm × 6 mm). **(D)** The model designed by CAD software, slicing CAD model, and the Code generation to control XYZ movement of the MtoBS. **(E)** Scaffold after 3D printing. **(F)** The scaffold is flexible, and a line spacing of 1,000–1,200 μm yields a similar tensile modulus to that of the auricle (Lee et al., 2014).

To make the 3D-printed scaffold more stable and complex, sacrificial layer technology was used to construct the support, with PEG as the sacrificial layer to stabilize the scaffold structure. At the same time, chondrocytes and adipocytes were implanted into corresponding positions in the scaffolds, including the cartilage and earlobe components for *in vitro* culture (Lee et al., 2014) (Figure 2). In recent years, in addition to commonly used materials such as PCL and PEG, the feasibility of using PU materials to print auricular cartilage has also been confirmed. Compared with the Medpor scaffold, PU has better histocompatibility and improved prospects for clinical applications.

### 4.2 3D-Printed Nasal Cartilage

Clinically, nasal cartilage defects have a significant impact on a person’s facial appearance and olfactory function. In nasal cartilage repair, autologous cartilage or bone is often implanted into the site that needs to be repaired. The materials commonly used for nasal scaffold transplantation include bone or cartilage obtained from the nasal septum, ribs, skull, and ear (Parkhouse and Evans, 1985). However, there is a risk of graft fracture and displacement when using a graft to repair the loss of nasal cartilage (Elik and Aktan, 2019; Cao et al., 2021). Therefore, the application of 3D printing in cartilage tissue engineering can provide a rational alternative for fabrication of nasal cartilage prostheses suitable for a given individual. Many researchers are committed to combining synthetic chemical materials and natural hydrogels into scaffolds using 3D printing. The use of PCL and various kinds of cellular hydrogels for scaffold fabrication has attracted much research interest in recent years (Table 2).

**TABLE 2:** Summary of 3D-printed nasal cartilage.

Material	Seed cells	Bioprinting Technology	Key points	References
Gelatin methacryloyl (GelMA)/Polyethylene glycol dimeth-acrylate (PEGDMA)/Gelatin	Chondrocytes	Extrusion printing	The engineered cartilage-like tissue construct was integrated with an electrochemical biosensing system to produce functional olfactory sensations	Jodat et al. (2020)
Gellan/Alginate/BioCartilage	Chondrocytes	Extrusion printing	A novel bio-ink for printing cartilage grafts was developed for use in 3D printing	Kesti et al. (2015)

#### 4.2.1 Preparation of Nasal Scaffolds With Hydrogel

**FIGURE 3:**
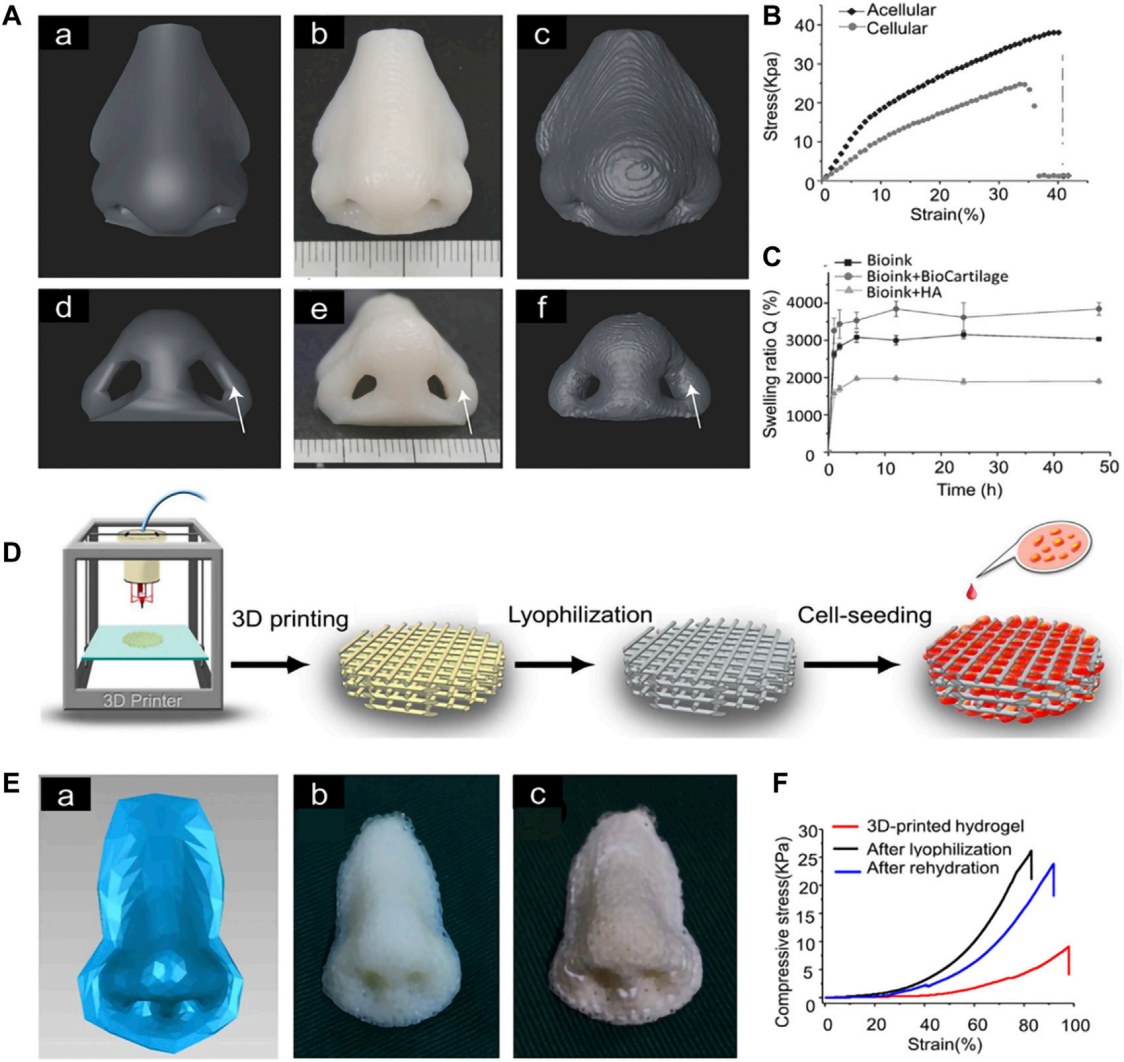
3D-printed nasal cartilage scaffold. **(A)** The 3D model for printing and comparison of its shape and volume after 2 weeks of swelling **(B)** The Young’s modulus of the acellular structure (E = 230 ± 7.0 kPa) was significantly higher than that of the cellular structure (E = 116 ± 6.8 kPa). **(C)** The equilibrium swelling rate of bio-ink + HA was significantly lower than that of bio-ink + biocartilage or bio-ink alone (Kesti et al., 2015). **(D)** Nasal cartilage scaffold was prepared by photocured 3D printing and lyophilization. **(E)** The scaffold printed according to the 3D model was lyophilized. **(F)** compression strength of the scaffold (Xia et al., 2018).

For 3D printing of nasal cartilage, researchers early on attempted to use pure hydrogel as the 3D printing material for nasal cartilage. However, performance of the printed nasal cartilage scaffolds can be increased by modification of the hydrogels. For example, swelling of the scaffolds can be increased significantly by adding extracellular matrix to the gel. When bio-ink for 3D printing is made of bio-cartilage, gellan gum, and alginate, the swelling rate of the scaffold is significantly greater than when it lacks bio-cartilage (Kesti et al., 2015). For scaffolds printed with hydrogel, the mechanical properties of the scaffolds can be improved by lyophilization (Xia et al., 2018) (Figure 3).

#### 4.2.2 Preparation of Nasal Scaffolds With Polymer Materials and Hydrogel

In order to make the mechanical properties of 3D-printed scaffolds approximate those of natural nasal cartilage, polymer and hydrogel were selected for 3D-printed nasal cartilage scaffolds. PCL is the most frequently used polymer. A multi-head disposition system can be applied to print PCL and alginate with both encapsulated cells and TGF-β. The cells in the printed scaffold maintained a high proliferation rate (Kundu et al., 2015). GELMA is also used to load cells and make nasal cartilage scaffolds with PCL. Moreover, 20% w/v gel is considered to be ideal for preparation of cartilage scaffold materials because of its rheology and improved biocompatibility (Ruiz-Cantu et al., 2020). To improve proliferation of chondrocytes on PCL scaffolds, dehydrated porcine nasal cartilage extracellular matrix was incorporated into the scaffold, which showed improved chondrogenic differentiation potential (Wiggenhauser et al., 2019). However, further research is urgently needed.

To improve nasal cartilage scaffolds, in addition to exploration of hydrogels, some have studied PCL as it mainly plays a role in maintaining shape. Filaments of carefully chosen mixtures of PCL and graphene are suitable for preparation of scaffolds by injection molding, which is conducive to growth of cartilage and can improve the mechanical properties of scaffolds (Rajzer et al., 2020). In printing cartilage scaffolds, the flexibility of the scaffold should also be considered in order to make the mechanical properties of the scaffold similar to those of nasal cartilage. Using an octahedral pore structure instead of a cube or lattice structure for 3D-printed nasal scaffolds can increase the flexibility of the scaffold and reduce the risk of prosthesis deformation and infection (Jung et al., 2014). A novel use of laser sintering to process PCL improved cell survival by controlling pore structure and promoting perfusion of the hydrogel (Zopf et al., 2015).

#### 4.2.3 Olfactory Restoration *via* a Novel Nasal Cartilage Scaffold

In addition to focusing on aesthetics in nose reconstruction, the sense of smell should also be considered. A recent study of nasal cartilage 3D printing reported the addition of an olfactory sensing device, which may represent a future direction in nose reconstruction research in which function is considered along with appearance (Jodat et al., 2020). Electrospinning of gelatin fibers on the surface of 3D printing scaffolds can be transformed into gelatin after heating, which helps to capture cells and increase cell adhesion after inoculation. Thus they can be well distributed in the porous PLLA scaffold (Rajzer et al., 2018).

### 4.3 3D-Printed Tracheal Cartilage Tissue

Cricoid hyaline cartilage maintains tracheal shape and guides air flow into and out of the lungs. In addition, the glands in the inner layer of cartilage can release cells and granules into the body by secreting mucus (Brand-Saberi and Schäfer, 2014). There are two kinds of common tracheal diseases: tracheomalacia caused by injuries to the tracheal wall, and tracheal stenosis caused by tumors, congenital defects, and other tracheal diseases (Rich and Gullane, 2012). Severe tracheomalacia leads to tracheal stenosis and can be life threatening. Procedures for assisting breathing include noninvasive ventilation, tracheostomy, and airway stenting (Janssen et al., 2021).

Treatment of tracheal tumors depends on tumor size. If the length of a tracheal resection is greater than 6 cm, it cannot be repaired by end-to-end anastomosis (Fabre et al., 2013); an implantable scaffold offers an alternative approach. When choosing a suitable scaffold, the mechanical strength, cell compatibility, and adhesion of the scaffold should be considered, but at present there is no perfect bionic scaffold (Jagdeep et al., 2017). In addition, for repair of tracheal injury in children, the scaffold must grow with age (Hamilton et al., 2015). Although biological 3D printing may be used to address these issues, hydrogel on its own as a scaffold material is unsuitable as it is too compliant to maintain an open channel for air flow through the trachea.

#### 4.3.1 Preparation of Tracheal Scaffolds Using Polymer Materials Combined With Hydrogel

Macchiarini et al. (2009) were the first to perform trachea replacement using an acellular allogeneic annular trachea. In combination with selecting human tissue to construct trachea, emerging 3D printing technology can provide patients with customized tracheal prostheses. Similar to auricular and nasal cartilage scaffolds, the material used in the initial study of tracheal printing was mostly PCL (Table 3) (She et al., 2021). An optimally structured PCL scaffold can be printed at 90°C with a heating nozzle (Ahn et al., 2019). Given the complexity of tracheal structure, the characteristics of each layer of a tracheal scaffold should be considered in its design. A 3D printing machine with a screw pump system and a pipe manufacturing controller has been used to print tracheal scaffolds composed of alginate combined with PCL and cells (Park et al., 2019). Porous PCL was located both inside and outside the scaffold, and the middle two layers of alginate + cells were separated by pore-free PCL.

**TABLE 3:** Summary of 3D-printed tracheal cartilage.

Material	Seed cells	Bioprinting Technology	Key points	References
PCL	Human bronchial epithelial cells (hBECs)/iPSC-derived mesenchymal stem cells (iPSC-MSCs)/iPSC-derived chondrocytes (iPSC-Chds)	Fused deposition system	Combined electrospinning technology and 3D printing technology to print tracheal cartilage	Kim et al. (2020)
PCL	Rabbit chondrocytes	Fused deposition system	The 3D-printed scaffold printed with PCL was loaded with cultured chondrocytes	Gao et al. (2017)
PCL	Goat auricular chondrocytes	Fused deposition system	PCL was used to print the cell scaffold, and the scaffold was cellularized	Xia et al. (2019)
PLLA	Rabbit chondrocytes	Fused deposition system	The 3D-printed scaffolds were seeded with chondrocytes obtained from autologous auricles	Gao et al. (2019)
PU	Mesenchymal stem cells (MSCs)	Liquid-frozen deposition manufacturing (LFDM)	A tracheal scaffold printed by Pu was tested *in vivo*	Hsieh et al. (2018)
PCL/Alginate	Rabbit bone marrow-derived mesenchymal stem cells (bMSC)/Epithelial Cells		Artificial trachea containing two cell types were fabricated by three-dimensional bioprinting	Bae et al. (2018)

**FIGURE 4:**
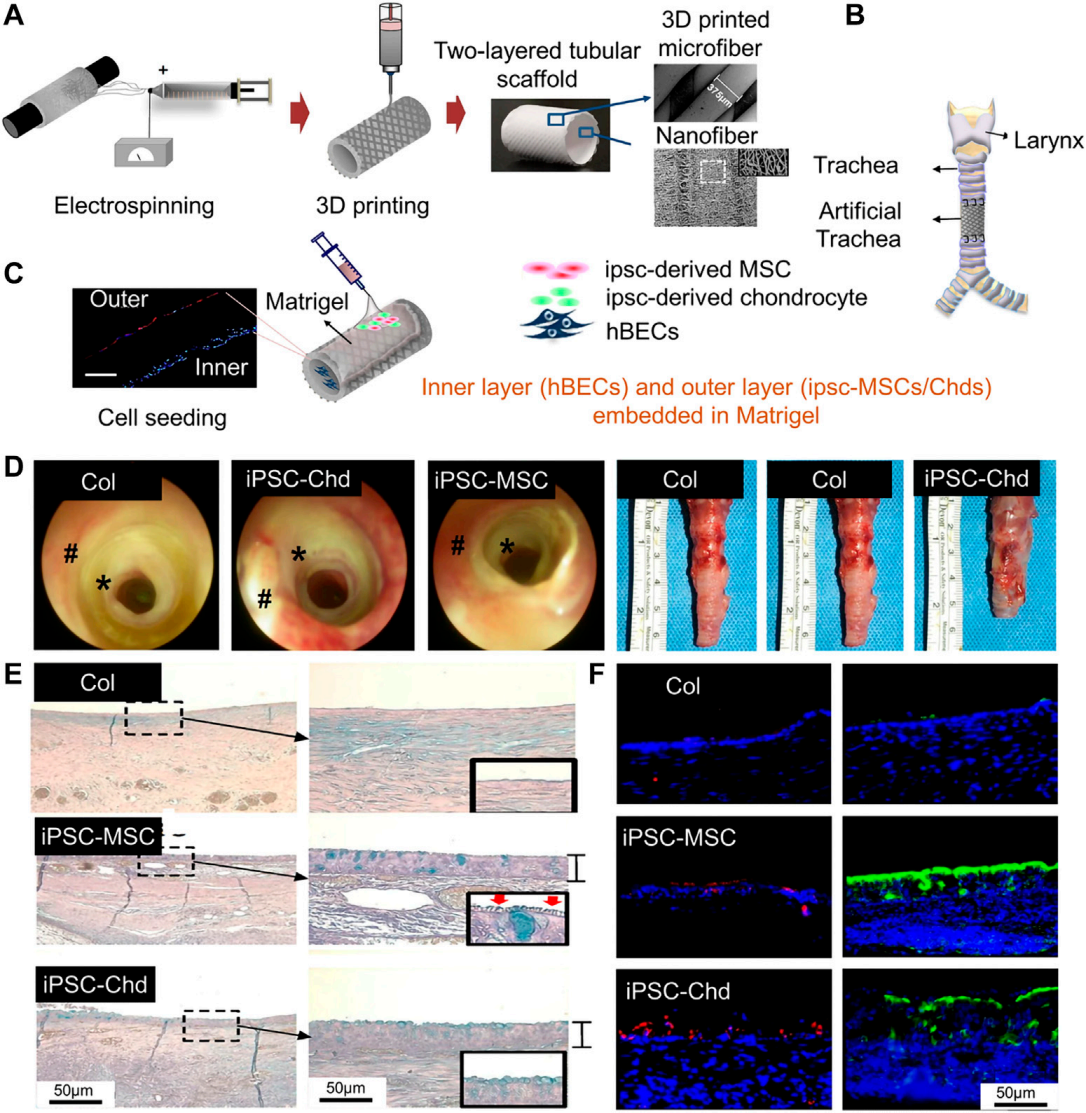
Process diagram for manufacturing a 3D tubular artificial tracheal scaffold. **(A)** A PCL tubular nanofiber layer (inner layer) prepared by electrospinning was wrapped with 3D-printed PCL fiber. **(B)** Tracheal transplantation and endoscopic analysis. **(C)** Induced pluripotent stem cell-derived chondrocytes (IPSC-CHDs) and human bronchial epithelial cells (hBECs) were separately seeded on the outer and inner layers of the scaffold. Each layer was identified by pkh-26 staining (red; IPSC-CHDs) and DAPI (blue; hBECs) staining (scale = 200 μm). **(D)** The endoscopic images at 4 weeks. **(E)** Alcian-blue staining showed mucus (blue) generated by regenerated tracheal epithelium 4 weeks after transplantation. (F) Immunofluorescence staining for β-tubulin and keratin-5 at the implant site (Kim et al., 2020).

The structure of PCL scaffolds can be improved by electrospinning technology, which can improve cell viability (Yu et al., 2021). For example, PCL nanostructured scaffolds were prepared as the inner layer of a tracheal scaffold using an electrostatic spinning technique, into which human bronchial epithelial cells (HBECs) were implanted. In addition, 3D-printed PCL scaffolds have been used as the outer layer of a tracheal scaffold. Induced pluripotent stem cell-derived mesenchymal stem cells (IPSC-MSCs) and induced pluripotent stem cell-derived chondrocytes (iPSC-Chds) were implanted into the outer layer of the scaffold. The outer layer of the scaffold maintained and supported the shape of the trachea. The nanostructure of the internal electrospun scaffold better simulates the extracellular matrix, which is more conducive to the growth of HBECs, enables rapid reconstruction of the mucosal layer, and can also reduce tracheal stenosis caused by incomplete reconstruction (Kim et al., 2020) (Figure 4). Comprehensive animal studies will be required before this method can be used for segmental bronchial transplantation.

The composite material obtained by adding chitosan to PCL has been used for electrospinning, which can reduce the diameter of electrospun fibers and improve the mechanical properties of the scaffold (Kim et al., 2021). To provide 3D-printed trachea with improved structure, the sacrificial layer technique has also been used in printing of tracheal cartilage. A sacrificial mold containing hollow trachea was constructed by 3D printing, and then PLCL was injected into the mold to generate an annular tracheal structure. Gelatin sponges loaded with transforming growth factor-β1 (TGF-β1) and chondrocytes were placed between the annular structures to promote formation of cartilage tissue (Park et al., 2015).

In addition to commonly used PCL materials, two kinds of PU materials with different compositions were used for 3D printing of the hard and soft segments of tracheal wall cartilage, and the printed 3D scaffolds were then implanted into nude mice for 6 weeks. The results showed that the maximum tensile stress, Young’s modulus, and elongation of the 3D tracheal scaffold were 4.6 MPa, 21.1 MPa, and 106.2%, respectively, and the MSCs on the scaffold could be induced to differentiate into chondrocytes (Hsieh et al., 2018). TPU has elastic properties appropriate for use as a tracheal support material. Researchers printed TPU scaffolds with wave and straight pattern micro-morphology. Electrospinning was used to cover the inner and outer surfaces of the TPU scaffold to improve its biocompatibility. Electrospinning on 3D-printed tracheal scaffolds can improve cell adhesion to the scaffolds and their elastic properties (Ahn et al., 2019).

#### 4.3.2 Blood Supply Improvement in 3D-Printed Tracheal Scaffolds

**FIGURE 5:**
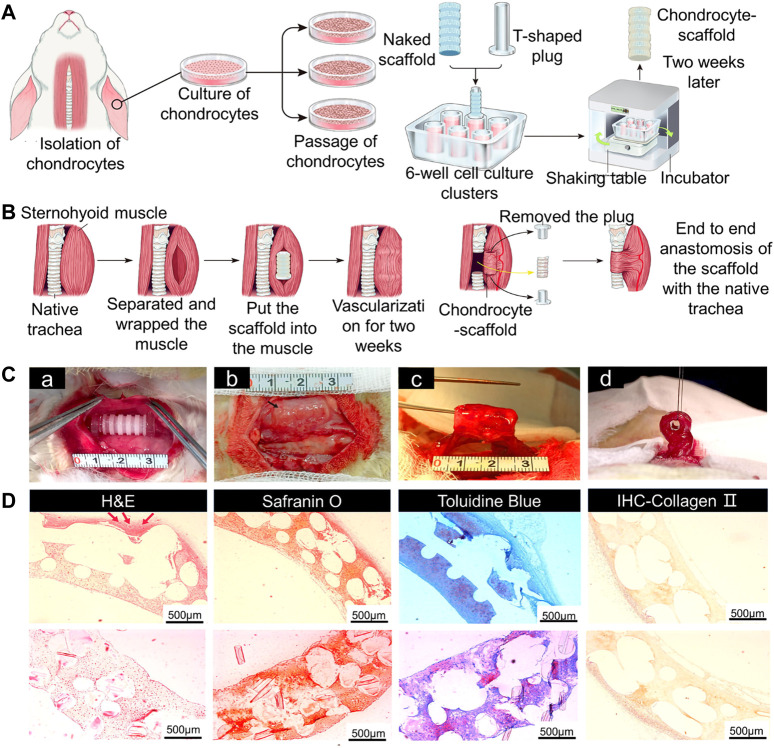
Tracheal cartilage scaffolds were made by 3D printing technology and tested *in vivo*. **(A)** Schematic diagram of 3D-printed PLLA tracheal scaffold. **(B)** Pre-vascularization and tracheal reconstruction of cellular scaffold constructs *in vivo*. **(C)** The scaffold was implanted into sternal muscle for pre-vascularization. A complete segmental tracheal organ unit with pedicle muscle flap was formed. **(D)** The images of H&E, safranin O, and toluidine blue staining, and IHC of type II collagen of chondrocyte-scaffold constructs. (Scale bar: 500 μm; red arrow: small blood vessels around the engineered trachea) (Gao et al., 2019).

**FIGURE 6:**
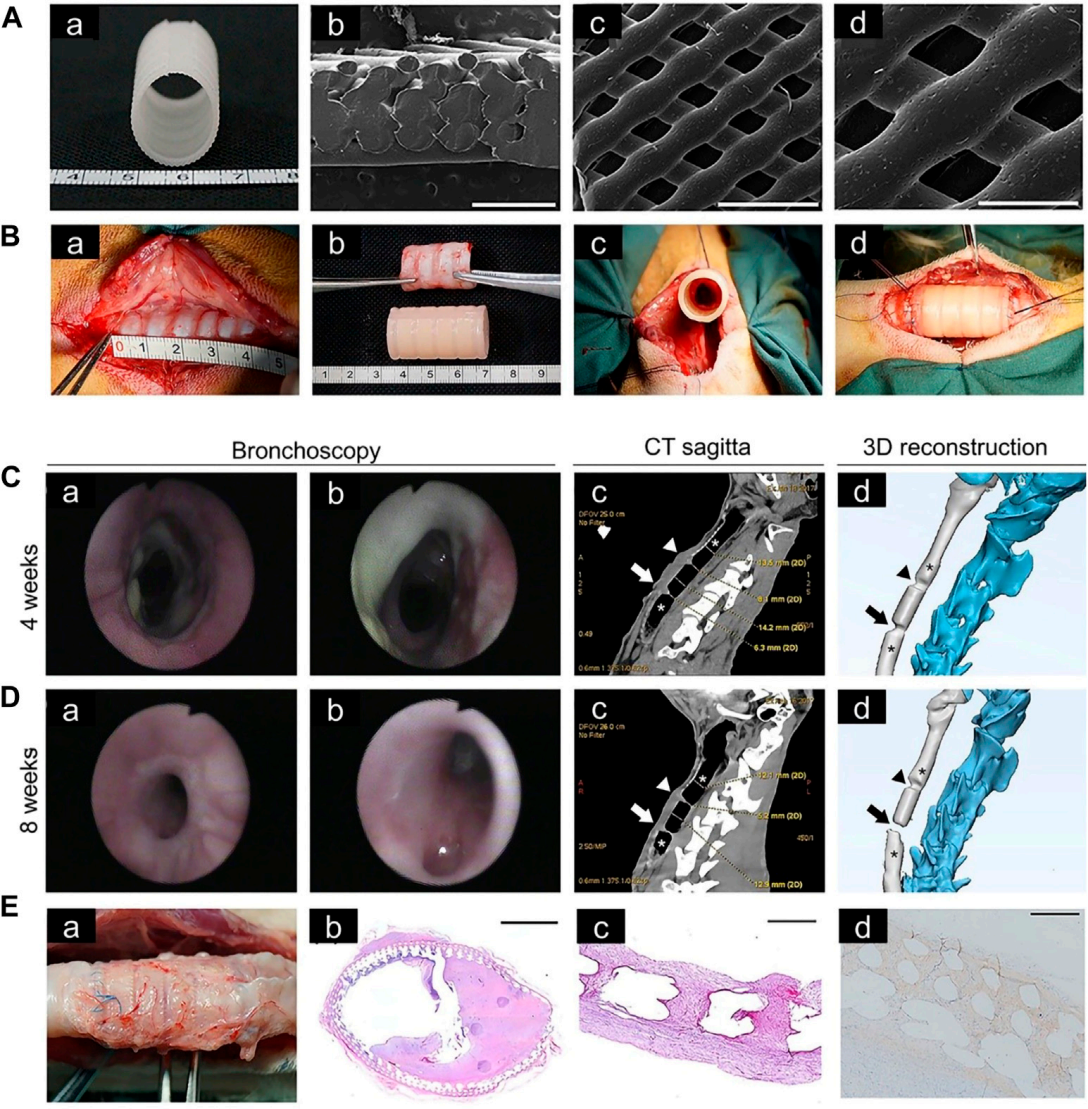
Tracheal cartilage scaffolds were made by 3D printing technology and tested *in vivo*. **(A)** Macroscopic **(a)** and SEM **(b–d)** images of 3D-printed PCL scaffold. **(B)** Implantation of a tracheal cartilage scaffold. **(C)** Four weeks after implantation, tracheal images were acquired at the upper, middle and lower anastomosis sites, respectively **(a,b)**. Computed tomography (CT) sagittal images and representative sections of 3D-printing reconstruction after 4 weeks, in which the narrow area represents the upper and lower anastomoses (arrow) **(c,d)**. **(D)** Bronchoscopy images and CT sagittal images after 8 weeks. **(E)** The scaffold of the experimental group was removed at the time of death **(a)**. Hematoxylin and eosin staining of scaffolds at low magnification **(b)**. Representative safranin O staining **(c)**. Representative collagen II staining **(d)** (Xia et al., 2019).

Blood vessels can promote growth of airway epithelium on the surface of 3D-printed scaffolds, therefore blood supply should be considered when designing tracheal cartilage scaffolds. As one approach to this problem, PLLA tracheal scaffolds were pre-cultured with chondrocytes *in vitro* and pre-vascularized *in vivo*, and then implanted with a muscle flap into tracheal defects in Manch rabbits (Gao et al., 2019) (Figure 5). The same group also transplanted 3D-printed trachea into sheep, which provided a basis for use of 3D-printed trachea in the clinic (Xia et al., 2019) (Figure 6).

Studies of segmental bronchial implants must also consider the problem of tracheal stenosis following transplantation. To solve this problem, some researchers use omentum to cover both ends of the prostheses to reduce complications, including luminal stenosis and mesh exposure (Teramachi et al., 1997). This method has also been used to facilitate tracheal scaffold blood flow (Masatsugu et al., 2014). In addition, by implanting a 3D-printed PCL scaffold into an animal’s omentum, it was shown that using the omentum as a 3D-printed scaffold bioreactor can not only improve blood flow and reduce the incidence of tracheal stenosis but also promote proliferation of tracheal epithelial cells (Park et al., 2018).

### 4.4 3D-Printed Meniscus Cartilage Tissue

The knee menisci are two fibrous cartilage discs in the joint that provide for shock absorption and load transmission. The lateral and medial menisci are semicircular and C-shaped, respectively (Chae et al., 2021). A meniscus can be divided into a white-white area, red-red area, and red-white area based on blood supply (Markes et al., 2020). Approximately ninety percent of meniscus injuries require meniscus removal because chondrocytes are not renewable. However, the incidence of osteoarthritis (OA) following meniscectomy is 7.4-fold higher than normal. Therefore, scaffolds with morphology and mechanical properties similar to menisci have attracted much research interest in recent years (Kwon et al., 2019; De Caro et al., 2020). For 3D printing of a tissue engineered meniscus, not only should morphological similarity be considered but also mechanical load-bearing and lubrication. A meniscus scaffold can be printed with PCL using FDM technology (Zheng-Zheng et al., 2016) (Table 4).

**TABLE 4:** Summary of 3D-printed meniscus cartilage.

Material	Seed cells	Bioprinting Technology	Key points	References
PCL	BMSCs	Fused deposition modeling (FDM)	PCL was printed into meniscal scaffolds with different pore sizes using the melt deposition technique	Zhang et al. (2016)
PCL/GelMA	Fibrochondrocytes	Bioscaffolder system (SYS + ENG, SalzgitterBad, Germany)	Agarose and gelatin methcrylate (GelMA) hydrogels were printed on the inside and outside of PCL scaffold, respectively	Bahcecioglu et al. (2019)
Gelatin/Alginate/CNF	Rabbit fibrochondrocytes (rFCs)	Extrusion printing	The gelatin-alginate bio-ink modified with cellulose nanofibers (CNF) has been verified for its feasibility to print meniscus	Luo et al. (2020a)
Silk fibroin/gelatin	Porcine fibrochondrocytes	Extrusion printing	The scaffold made of silk fibroin and gelatin has good performance after freeze-drying	Bandyopadhyay and Mandal (2019)
Meniscus extracellular matrix (MECM)/sodium alginate	Meniscal fibrochondrocytes (MFCs)	Fused deposition modeling (FDM)	The scaffold was made of MECM-alginate bio-ink and 3D-printed PCL.	Chen et al. (2019a)
Decellularized meniscal extracellular matrix (dECM)/PU/PCL	BMSCs	FDM/Extrusion	Hydrogel made from decellularized extracellular matrix and a mixture of PCL and PU was used to make meniscal scaffolds	Chae et al. (2021)

#### 4.4.1 Preparation of Meniscus Scaffolds With Hydrogel

Among the hydrogels used in meniscus scaffolds, GelMA has superior properties for printing and can maintain higher fidelity (Daly et al., 2016). In addition, gelatin-alginate bio-ink containing cellulose nanofibers (CNF) has been shown to be useful for printing menisci (Luo W. et al., 2020). The mechanical properties of scaffolds made of silk fibroin and gelatin were greatly improved and made more similar to menisci by freeze-drying and cross-linking with EDC (1-ethyl-3-(3-dimethylamino propyl) carbodiimide hydrochloride) and NHS (N-hydroxysuccinimide) (Bandyopadhyay and Mandal, 2019). ECM extracted from menisci mixed with cells and growth factors is promising for use in meniscus tissue engineering (Romanazzo et al., 2017). Hydrogel produced by decellularized ECM and sodium alginate can be combined with PCL to prepare meniscal scaffolds (Chen M. et al., 2019). There are differences in the properties of ECM extracted from the inner and outer menisci: inner meniscus ECM can promote chondrogenesis of fat pad-derived stem cells, while outer meniscus ECM can promote development of cells with thinner and more fibroblast-like phenotypes (Romanazzo et al., 2017).

#### 4.4.1 Preparation of Meniscus Scaffolds With Polymer Materials and Hydrogel

For meniscus scaffolds made of PCL, scaffold pore size influences ECM production, cell behavior, biomechanics, and hence successful repair. A scaffold with an average pore size of 215 μm not only has good tensile and compressive properties but can also promote proliferation and differentiation of MSCs (Zhang et al., 2016). A meniscus scaffold has been constructed of agarose and gelatin methacrylic acid (GelMA) hydrogels printed on both sides of a PCL scaffold. Production of glycosaminoglycans and type II collagen was promoted by agarose, while GelMA promoted production of type I collagen (Bahcecioglu et al., 2019). In addition, a scaffold made by placing silk fibroin on both sides of a 3D-printed PCL mesh also has good biocompatibility and is more suitable for tissue infiltration and blood vessel formation (Cengiz et al., 2019).

**FIGURE 7:**
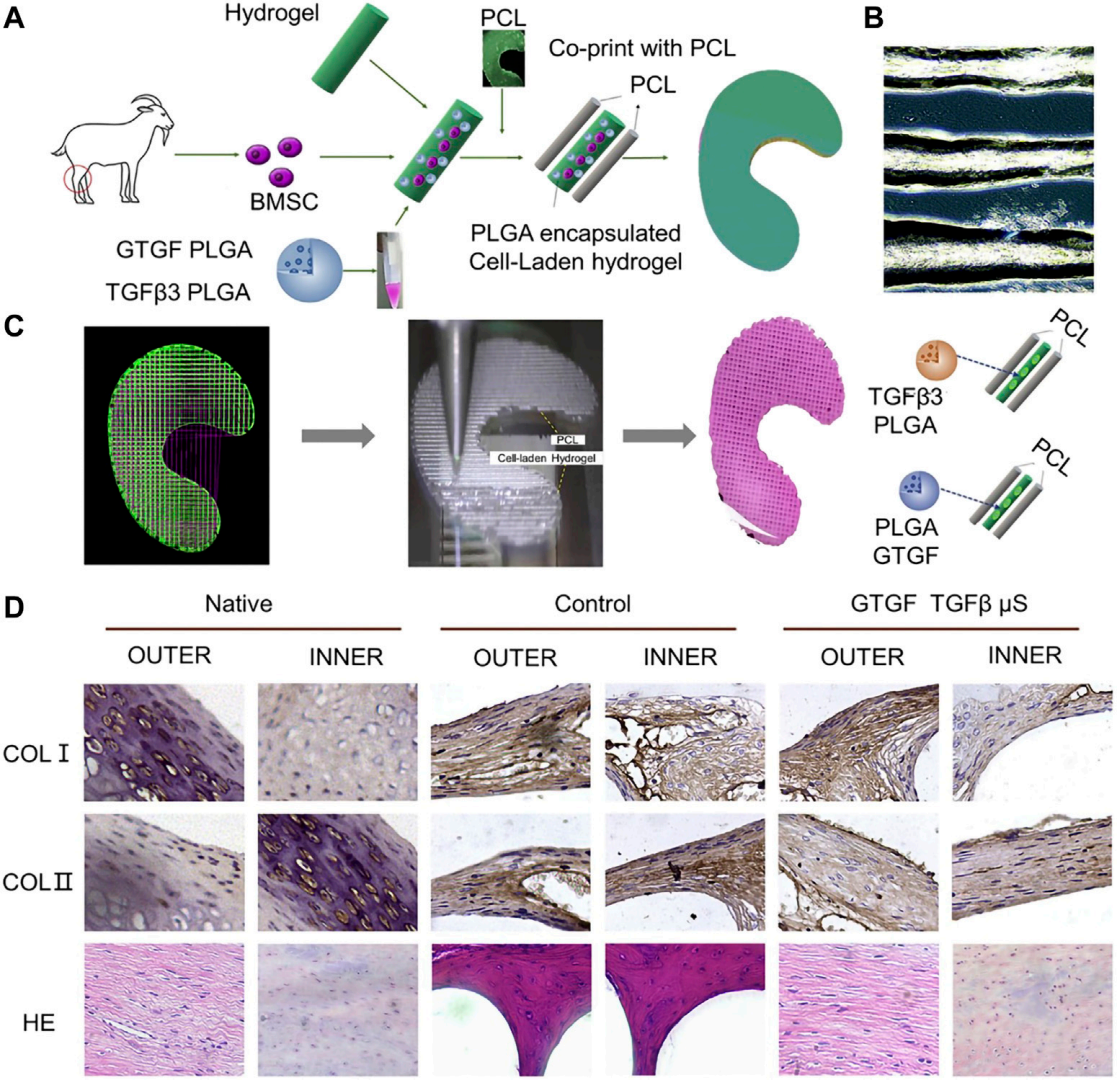
PCL and PLGA encapsulated cell-laden hydrogel was used to make a meniscus scaffold by 3D printing technology. **(A)** Schematic diagram of the design of a bioprinted composite material study for regeneration of a goat meniscus. **(B)** PLGA-encapsulated hydrogels containing MSCs cells exhibited good printability. **(C)** CAD model of the meniscus for 3D printing. The cell hydrogel and PCL were printed into 3D meniscus by 3D printing technology. **(D)** Zone-specific matrix phenotype analysis in engineered versus native tissue (Sun et al., 2020).

PLGA, in addition to being combined with hydrogel and ECM as a carrier of cells, can itself be loaded with cells and growth factors. For instance, PLGA microparticles loaded with MSC cells and TGFβ3 or CTGF can be deposited on a PCL scaffold to make a meniscus scaffold. Activity and proliferation of BMSCs were similar when loaded into seven types of hydrogel (Sun et al., 2020) (Figure 7). In addition to growth factors that promote cell proliferation, BMSC-specific-affinity peptide can enhance recruitment and retention of endogenous BMSCs when placed on the surface of composite scaffolds made of SF and PCL, thus reducing cell loss and enhancing scaffold chondrogenicity (Li et al., 2020). In addition to the materials discussed above, silicone can be made into a meniscus scaffold with excellent biocompatibility by using the method of heat-cured extrusion (Luis et al., 2019).

## 5 Future Prospects

To date there have been several technological advances in 3D printing of tissue-engineered cartilage, and 3D printing of irregularly shaped cartilage has evolved from simple morphological imitation to sophisticated biological tissue engineering. However there is still a need for in-depth research and continuous innovation to improve the effectiveness of complex tissue-engineered cartilage scaffolds.

### 5.1 Optimization of Technology and Materials Required for 3D Printing

For 3D printing of irregularly shaped cartilage scaffolds, further optimization of 3D printers is needed to increase the range of applications. For example, achieving more precise dynamic control of printing temperature for materials that require high or low temperature would allow for combining polymers and cell-loaded hydrogels into a single scaffold.

In terms of the choice of synthetic macromolecular polymers, the main problem to be solved is whether the hardness of the resulting material is close to that of natural cartilage. PU has been shown to have superior elastic properties, which may make it the first choice among cartilage materials in the future. For selection of cell carriers such as hydrogels, we need to identify biomaterials that can better promote cell viability. At the same time, it is hoped that some drugs, such as antibiotics or growth factors, can be mixed with hydrogels or evenly coated on 3D-printed scaffolds to reduce inflammation or promote cartilage regeneration, respectively. In addition, the material properties of synthetic macromolecular polymers and hydrogels can be modified to increase cell adhesion.

### 5.2 Optimization of 3D-Printed Cartilage Scaffolds

Research on printing of irregularly shaped 3D cartilage scaffolds has mainly focused on simulation of morphology, while only a few studies, such as those concerned with tracheal scaffolds, have considered optimization of function. For example, improving blood circulation in and around cartilage may increase biocompatibility of the overall scaffold, which may be achieved by implanting 3D-printed cartilage with embedded blood vessels into cartilage repairs. In the future, it may be possible to print auricular and nasal cartilage scaffolds and skin at the same time using 3D printing technology. For printed tracheal cartilage, materials that can adapt to airflow should be further studied. As for the meniscus, because its surface is smooth in the physiological state, this should also be true of the 3D-printed scaffold.

### 5.3 4D Printing

Based on 3D printing research, 4D printing is gradually being applied in the field of cartilage tissue engineering. The fourth dimension in 4D printing results from changes in conditions, e.g., light, electromagnetic fields, water, or temperature. Some materials with shape memory can restore their original shape under certain conditions (Wu et al., 2018). On this basis, the shape of the hydrogel loaded with a drug could be made to change after being implanted. This technique can not only alter the shape of the scaffold but also slow drug release. Hopefully, complex structural shapes can be optimized by 4D printing. In the future, it may also be possible to inject cells into the closed spaces when the 4D-printed scaffold is deformed in order to prevent loss of cells.

### 5.4 Applications *In Vivo* and in Clinical Trials

At present, although many kinds of research are devoted to 3D-printed cartilage scaffolds, most lack sufficient clinical trials and are not mature enough to be used in the clinic. A perfect cartilage scaffold needs *in vitro* and *in vivo* experiments to verify its feasibility. If it is to be used in the clinic, follow-up clinical trials are also essential. Therefore, there is still a great need for further research on 3D printing of tissue-engineered cartilage. Nevertheless, some 3D-printed scaffolds are expected to replace implant materials currently on the market.

## 6 Conclusion

3D-printed cartilage has been successfully utilized in various medical fields. This review discusses in detail selection and preparation of various irregularly shaped cartilage scaffolds, including auricle, nasal, tracheal, and meniscus. Using 3D printing technology, scaffolds printed with biocompatible high molecular-weight polymer materials can be combined with hydrogel and chondrocyte matrix to produce ideal irregularly shaped cartilage scaffolds. In addition, we look forward to optimization of 3D printing technology and the materials required for 3D printing. 3D printed cartilage scaffolds can be optimized by increasing blood supply, adding the concept of 4D printing, and increasing research *in vivo* which moves on to clinical trials. We are hopeful that these considerations will be integrated into future research.
